# Evaluation on the efficacy and safety of CyberKnife stereotactic radiotherapy for brainstem gliomas

**DOI:** 10.3389/fonc.2026.1730758

**Published:** 2026-03-27

**Authors:** Penghui Luo, Tingting Wei, Youling Zhang, Guimei Wang, Wenchao Yuan, Min Kang, Zuping Lian

**Affiliations:** 1 Department of Radiation Oncology, The First Affiliated Hospital of Guangxi Medical University, Nanning, Guangxi, China; 2 Department of Radiation Oncology, The Third Affiliated Hospital of Guangxi Medical University, Nanning, Guangxi, China; 3 Department of Medical Oncology, Ruikang Hospital Affiliated to Guangxi University of Chinese Medicine, Nanning, Guangxi, China; 4 Key Laboratory of Early Prevention and Treatment for Regional High Frequency Tumor (Guangxi Medical University), Ministry of Education, Nanning, Guangxi, China; 5 Guangxi Key Laboratory of Immunology and Metabolism for Liver Diseases, Nanning, Guangxi, China; 6 State Key Laboratory of Targeting Oncology, Guangxi Medical University, Nanning, Guangxi, China

**Keywords:** brain stem, CyberKnife, glioma, radiosurgery, stereotactic radiation therapy

## Abstract

**Background:**

Brainstem glioma (BSG) is a rare neoplasm characterized by short survival and high mortality. Thus far, treatment efficacy for this tumor has remained limited, highlighting the critical demand for novel and effective therapeutic strategies. The utilization of CyberKnife Stereotactic Radiation Therapy(CyberKnife-SRT) for BSG may render favorable outcomes. Nevertheless, robust clinical data remain limited at present. Therefore, this study was designed to assess the efficacy and safety of CyberKnife-SRT in the treatment of BSG.

**Methods:**

A retrospective analysis was conducted using clinical data from patients treated with CyberKnife-SRT for BSG between August 2009 and July 2024. Demographic characteristics and treatment parameters were extracted. Overall Survival (OS) was defined as the interval from initial diagnosis to death from any cause or the last follow-up; survival curves were estimated with the Kaplan–Meier method, and inter-group differences were evaluated using the Log-rank (Mantel-Cox) test. Univariate Cox proportional-hazards models were fitted to assess the association between each candidate variable and the risk of death. Subsequently, variables were selected by backward stepwise elimination based on Akaike’s information criterion (AIC), and a multivariate Cox proportional-hazards model was constructed to identify factors independently associated with mortality. And the incidence of adverse reactions of the patients were analyzed.

**Results:**

A total of 54 BSG patients treated with CyberKnife-SRT were enrolled, with a median follow-up of 71 months (Range: 3.03 - 182.97 months). Median Overall Survival (mOS) for the entire cohort was 20.9 months (95% CI 15.7 - 67.6). Median Progression-free Survival (mPFS) was 13.7 months(95% CI 8.83 - 20.08). The 1-year and 5-year OS rates were 83.39% and 32.74%, respectively. The 1-year and 5-year PFS rates were 57.02% and 21.36%. Univariate Cox regression analysis revealed that tumor volume > 8 cm³ (HR 3.04, 95% CI 1.61 - 7.97, p = 0.024), age 3–17 years (HR 2.27, 95% CI 1.04 - 4.96, p = 0.039), and age 51–69 years (HR 5.47, 95% CI 1.76 - 17.02, p = 0.003) were significantly associated with an increased risk of death, whereas Karnofsky Performance Status (KPS) score ≥ 70 was the only protective factor identified (HR 0.39, 95% CI 0.19 - 0.82, p = 0.013), while surgery, chemotherapy and targeted therapy had no significant impact. Multivariate Cox regression analysis showed that, compared with the 18–50-year age group, the 51 - 69-year age group had a significantly higher risk of death (HR = 6.69, 95% CI 2.03-22.00, p = 0.002). Regarding safety, 17 (31.48%) patients developed radiation-induced brain edema, and 4 (7.41%) patients had obstructive hydrocephalus. All conditions improved after active treatment. No radiation necrosis or radiotherapy-related deaths were observed.

**Conclusions:**

Indirect comparison with historically reported data indicates that CyberKnife-SRT is associated with prolonged survival in patients with BSG, with manageable treatment-related toxicities. These results suggest a favorable benefit-risk profile for CyberKnife-SRT in this patient population.

## Introduction

Brainstem glioma (BSG) accounts for approximately 2.5% of all malignant neoplasms of the brain and central nervous system (CNS) and is considered a relatively rare entity (1). Clinical studies on BSG are limited. As the vital neural center of the human body, the brainstem is indispensable; its impairment constitutes an immediate life-threatening risk, and Magnetic Resonance Imaging (MRI) has become the main diagnostic method (2). Radical surgery is extremely difficult, biopsy is also very challenging, and postoperative complications are severe (3). The most prevalent type of BSG, namely diffuse intrinsic pontine glioma (DIPG), is nearly inoperable and has an extremely poor prognosis, with a median survival of less than one year (4).

Radiotherapy has consistently been the principal modality for treating BSG, and its role in the management of DIPG remains irreplaceable (4, 5). Conventional radiotherapy, delivered in multiple fractions, is often associated with poor patient compliance. It provides inadequate sparing of normal tissues, making it difficult to escalate the biologically effective dose and resulting in significant radiation-induced injury. Stereotactic radiotherapy (SRT) typically employs hypofractionated regimens of a few fractions, delivering a high biologically effective dose while causing relatively minor damage to normal tissues (6, 7). Theoretically, it offers notable radiobiological advantages for BSG. Nevertheless, current evidence has not yet conclusively demonstrated the superiority of SRT in the treatment of BSG (6).

CyberKnife is an advanced frameless, image-guided, robotic SRT platform that markedly improves patient comfort (7). It delivers sub-millimetre geometric accuracy, provides real-time tumour tracking and dynamic error correction, and thus maximally spares surrounding critical structures while reducing the risk of radiation-induced normal-tissue injury (8, 9). Hypofractionated, high-dose schedules shorten overall treatment time and significantly enhance compliance.8 Consequently, CyberKnife-based SRT (CyberKnife-SRT) may represent a preferred option for brainstem glioma at present; however, robust clinical data are scarce and its efficacy and safety remain incompletely defined.

This retrospective study aims to systematically evaluate the efficacy, safety, and prognostic factors of CyberKnife-SRT to determine its role as a preferred treatment for BSG.

## Patients and methods

### Patient characteristics

The medical records of 124 patients with brainstem tumors who were treated with CyberKnife-SRT from August 2009 to July 2024 at the Ruikang Hospital Affiliated to Guangxi University of Chinese Medicine were reviewed. Based on the inclusion and exclusion criterias, rigorous screenings were carried out, and eventually a total of 54 patients with BSG were incorporated into this study. BSG patients are a mixed group consisting of both children and adults. Eight patients (14.81%) received histopathological diagnoses, including 4 cases of low-grade glioma and 4 cases of high-grade glioma. This study primarily relied on imaging for diagnosis, There are no restrictions on specific molecular pathological types. This approach was adopted because BSG are relatively rare, the limited number of cases, combined with the deep-seated tumor location and the abundance of surrounding cranial nerve nuclei, makes biopsy or surgery prohibitively risky, rendering pathological tissue acquisition difficult.

Inclusion Criteria: (1)The age ranged from 3 to 70 years. (2)Availability of cranial MRI scans performed within one month prior to treatment, including T1-weighted, T2-weighted, T1-enhanced, and fluid-attenuated inversion recovery (FLAIR) sequences, that meet the imaging diagnostic criteria. (3) All images were independently reviewed by two radiologists, each with at least five years of experience in neuroimaging diagnosis. Any discrepancies in their interpretations were resolved through discussion to reach a consensus. (4) The clinical data encompassed the patient’s gender, age, diagnosis, lesion location, treatment process, tumor volume, prescribed dose, and radiotherapy side effects.(5) All patients underwent CyberKnife-RT.(6) There were imaging re-examination results after radiotherapy.

**Figure 1:**
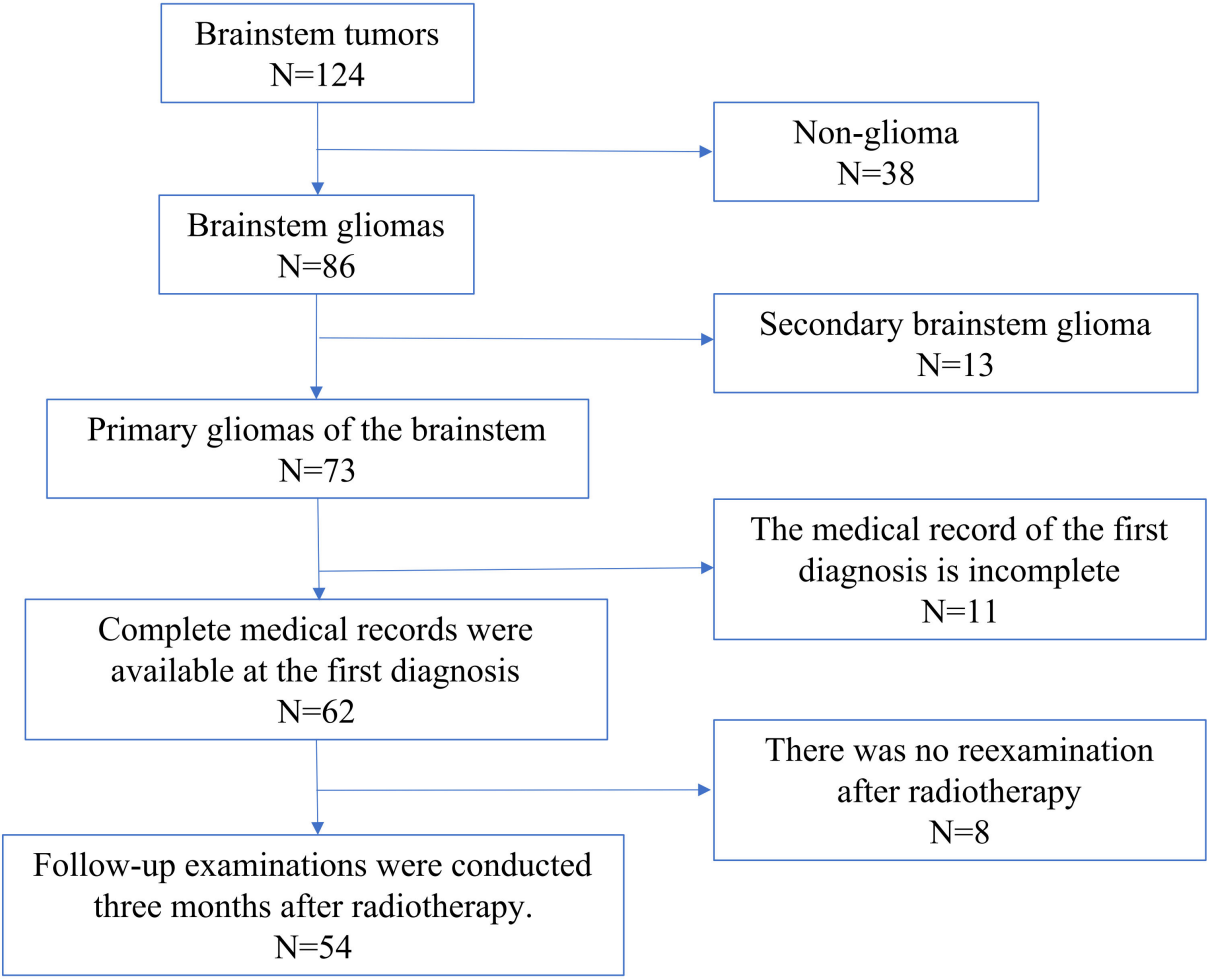
Flowchart for the screening of patients with brainstem glioma (BSG).

Exclusion Criteria: (1) Incomplete medical records. (2) No re-examination after radiotherapy. (3) History of other brain tumors. (4) Atypical imaging findings or inability to establish a definite diagnosis. (5) Did not receive CyberKnife-RT or experienced treatment interruption (**Figure 1**).

The imaging diagnostic criteria for BSG were located in the midbrain, pons, and/or medulla oblongata. On MRI, they appeared hypointense or isointense on T1-weighted and hyperintense on T2-weighted and FLAIR sequences. DIPG were characterized by diffuse pontine expansion, with abnormal T2 signal occupying ≥50% of the axial cross-sectional area of the pons and encasing the basilar artery. Contrast enhancement was either absent or mild; Focal gliomas presented with relatively well-defined borders and could be accompanied by cystic components and nodular enhancement; High-grade gliomas exhibited signs of significant heterogeneous enhancement, as well as necrosis and hemorrhage.

### Radiation therapy

Accurate radiotherapy positioning was accomplished via the combination of computed tomography (CT) plain scan and enhanced scan, with the slice thickness of the scan set at 1.25 mm. The gross tumor volume (GTV) was precisely delineated through comprehensive analysis of CT and MRI images. The total radiotherapy dose was 25–28 Gy, which was divided into 5–6 fractions, with the median prescription dose line ranging from 59% to 88%. Based on the α/β value of 3 for BSG and 2 for the brainstem, the biological effective dose (BED) was computed by employing the linear quadratic (LQ) model.

The volume of the brainstem (excluding medulla) exposed to a maximum dose of 23 Gy didn`t exceed 0.5 cm³, with the maximum did not exceed 31 Gy. For the spinal cord and medulla, the volume receiving a maximum dose of 22 Gy was less than 0.35 cm³, with the highest permissible dose was set at 28 Gy. In the case of the optic pathway, the volume subjected to a maximum dose of 23 Gy was below 0.2 cm³, and the maximum allowable dose was 25 Gy.

### Adjuvant therapic regimen

According to the specific condition of the patient, decision was made to employ adjuvant therapy or not. All anti-tumor drug were arranged to be conducted after the completion of radiotherapy. The specific medication regimens were as follows: Temozolomide was utilized for chemotherapy. The dosage in the first cycle was set at 150 mg/m² per administration, with continuous administration for five days and each 28 days constituting a cycle. If no toxic reactions of grade 3 or above were observed during the treatment process, the dosage was adjusted to 200 mg/m² in the subsequent cycles. If toxic reactions of grade 3 or above occurred, the dosage was decreased by 50 mg/m². When the dosage dropped below 100 mg/m², the drug was terminated. The dosage of Bevacizumab was 5 to 10 mg/kg per administration, with administration occurring once every 2 to 3 weeks.

### Follow-up and reexamination

MR or CT was used for follow-up examinations. Within one year subsequent to the completion of CyberKnife-SRT, a follow-up or reexamination was to be carried out every three months. After that one follow-up visit per year was required. In the event that the patient presented with uncomfortable symptoms, they would visitted a doctor immediately and underwent corresponding treatment.

The efficacy evaluation was conducted in accordance with the Response Evaluation Criteria in Solid Tumors (RECIST Version 1.1) (10) and accomplished via MRI or CT.

### Statistical analysis

Candidate prognostic factors were selected based on a review of the literature and their clinical relevance, including age, gender, Karnofsky Performance Status (KPS) score, tumor location, peritumoral drainage, local invasion, surgical resection, hydrocephalus, pharmacotherapy, and tumor volume. According to literature, clinical significance, and sensitivity analysis, among other multi-step methods, the optimal cut-off values for age, KPS score, and tumor volume are determined. Univariate Cox regression analysis was initially performed to evaluate the individual association of each variable with overall survival. To avoid overfitting and ensure model stability, variable selection for the multivariate Cox model was guided by the Akaike Information Criterion (AIC) using a backward stepwise procedure. The final model was determined by balancing the AIC, the statistical significance observed in univariate analyses, and the events-per-variable (EPV) principle. An EPV of ≥ 10 was considered sufficient to obtain reliable estimates, where EPV is defined as the total number of outcome events divided by the number of covariates (or parameters) included in the multivariate model. The overall missing data rate in this study was not included in the survival analysis, univariate analysis, or multivariate analysis.

The datas were statistically analyzed by the survival and survminer packages within R 4.4.1 statistical software. Overall survival time(OS) was delineated as the temporal interval from the initial diagnosis to the patient’s decease or the last follow-up. Progression-free survival time(PFS) was defined as the time from the first diagnosis to the first disease progression or the last follow-up.

Survival analysis, as the principal analytical approach, was employed to assess the pertinent factors influencing the prognosis of patients. The Kaplan-Meier approach was employed to estimate median OS (mOS) and median PFS (mPFS), and to compute the survival rates. The log-rank test was adopted to compare the discrepancies in survival rates. The univariate Cox regression models were utilized to analyze the influence of single variables on survival outcomes. The Cox proportional hazards model was used to analyze the relationship between multiple variables and survival. All statistical tests were bilateral, and a P value < 0.05 was regarded as the criterion for statistical significance.

The treatment-related toxicities were assessed in accordance with the Common Terminology Criteria for Adverse Events version 4.0 (CTCAE v4.0) (11). Following radiotherapy, if any novel symptoms or aggravation of neurological symptoms emerged, but magnetic resonance imaging (MRI) revealed no radiological disease progression, the amentioned situations were regarded as being related to the treatment.

## Results

### Demographics

The median age of the group was 26.5 years (rang: 3–69 years). 70.37% patients were males, 29.63% were females. The median KPS at diagnosis was 70 (rang: 30-90). 66.67% BSG were located in pons. Among them, 22.22% invaded the tissues outside brainstems. All patients sought medical attention due to clinical symptoms. And the most common symptoms and signs were cranial nerve palsy and motor disorders. 14.81% of BSG received a pathological diagnosis via surgery or biopsy. 1 case received radiotherapy after postoperative recurrence. The incidence of obstructive hydrocephalus was 16.67%, of which 9.26% occurred before radiotherapy and 7.41% occurred after radiotherapy (**Table 1**).

**Table 1:** Fundamental Information of Patients.

Feature	Case(%)	Feature	Case(%)
age(year)	26.5(3-69)	Symptoms and Signs	
3-17	22(40.74%)	Cranial nerve palsy	31(57.41%)
18-50	25(46.30%)	Motor disorder	29(53.70%)
51-69	7(12.96%)	Fatigue	26(48.15%)
sex		Dizziness/Headache	16(29.63%)
Male	38(70.37%)	Nausea/Vomiting	10(18.52%)
Female	16(29.63%)	Reduced sensation	8(14.81%)
KPS(score)		Pathology	8(14.81%)
90	1(1.85%)	Postoperative	6(11.11%)
80	17(31.48%)	Pilocytic astrocytoma	3(5.56%)
70	24(44.44%)	Grade II astrocytoma	1(1.85%)
60	10(18.52%)	Diffuse glioma	2(3.70%)
50	1(1.85%)	Post-biopsy	2(3.70%)
30	1(1.85%)	Anaplastic astrocytoma	1(1.85%)
Number of tumors		Diffuse midline glioma	1(1.85%)
1	46(85.19%)	Tumor invasion outside	12(22.22%)
>1	8(14.81%)	Tumor location	
Obstructive hydrocephalus	9(16.67%)	Midbrain	8(14.81%)
Before radiotherapy	5(9.26%)	Pons	36(66.67%)
After radiotherapy	4(7.41%)	Medulla oblongata	10(18.52%)

### Treatment

A total of 30 (55.56%) patients underwent radiotherapy alone, 5 (9.26%) underwent postoperative radiotherapy, 1 (1.85%) received radiotherapy due to postoperative recurrence. Additionally, 23 (42.59%) patients received Temozolomide chemotherapy, and 14 (25.93%) received Bevacizumab targeted therapy, among whom 13 (24.07%) received both treatments concurrently. Furthermore, 6 (11.11%) patients underwent ventricular drainage. The median target volume was 19.29 cm³ (rang: 0.4-79.13 cm³). The median total radiotherapy dose was 25 Gy (rang: 25–28 Gy). The median BED was 66.67Gy (rang: 66.67-77.92 Gy). The median prescription dose line was 66% (rang: 59% - 88%). And the median number of fractions was 5 (rang: 5 - 6) (**Table 2**).

**Table 2:** Therapeutic approaches.

Therapeutic approaches	Case(%)
**Radiotherapy**	54(100%)
Median Target Volume (cm³)	19.29 (0.4 - 79.13)
Total dose (Gy)	
25	51(94.44%)
27	1(1.85%)
27.5	1(1.85%)
28	1(1.85%)
Median BED (Gy)	66.67 (66.67 - 77.92)
Number of Fractions (times)	
5	53(98.15%)
6	1(1.85%)
Median prescription dose line	66%(59-88%)
Simple radiotherapy	30(55.56%)
Postoperative radiotherapy	5(9.26%)
Postoperative recurrence radiotherapy	1(1.85%)
**Surgery**	5(9.26%)
Chemotherapy (Temozolomide)*	23(42.59%)
Monotherapy chemotherapy	10(18.52%)
Targeted therapy (Bevacizumab)*	14(25.93%)
Monotherapy targeted	1(1.85%)
**Chemotherapy + targeted**	13(24.07%)
Ventricular drainage**	6(11.11%)
Before radiotherapy	3(5.56%)
After radiotherapy	4(7.41%)

*A total of 13 patients concurrently received both chemotherapy (temozolomide) and targeted therapy (bevacizumab). **One patient underwent ventricular drainage both prior to and subsequent to radiotherapy. Abbreviation: BED, biologically effective dose.

### Post-radiotherapy symptomatology evolution

After radiotherapy, symptomatic amelioration was observed in 46(85.19%) cases, symptomatic stability was noted in 3(5.55%) cases, and symptomatic exacerbation occurred in 5 (9.26%) cases. Notably, the amelioration was particularly significant in terms of motor dysfunction, headache, vomiting, and sensory reduction (**Table 3**).

**Table 3:** Post-radiotherapy symptomatology evolution.

Symptom change	N (%)
Symptomatic amelioration	46(85.19%)
Symptomatic stability	3(5.55%)
Symptomatic exacerbation	5(9.26%)

### Survival

**Figure 2:**
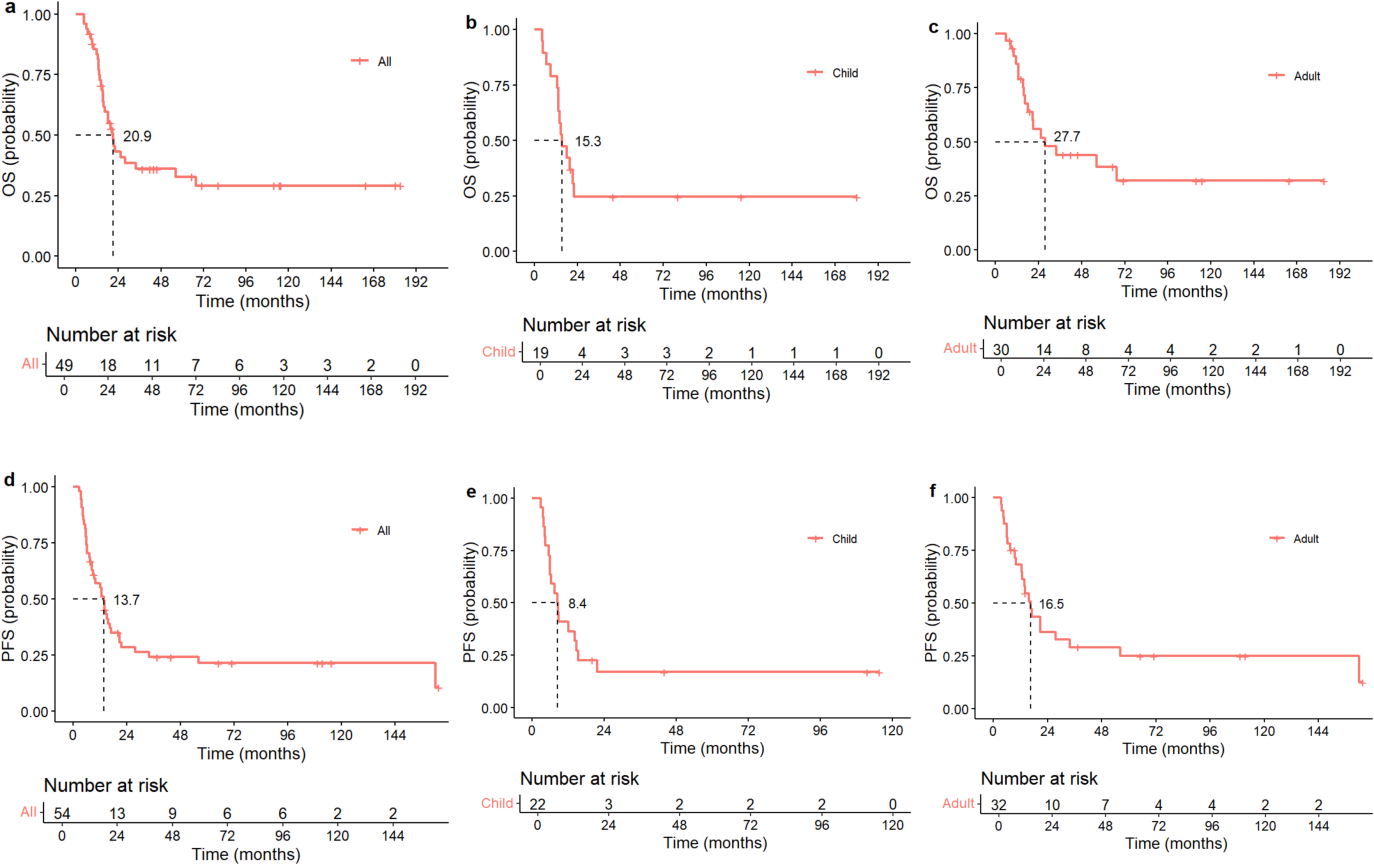
Kaplan-Meier survival estimates: **(a)** mOS = 20.9 months for the entire cohort; **(b)** mOS = 15.3 months for paediatric patients; **(c)** mOS = 27.7 months for adults patients; **(d)** mPFS = 13.7 months for the entire cohort; **(e)** mPFS = 8.4 months for paediatric patients; **(f)** mPFS = 16.5 for adults patients. Abbreviation: mOS, Median Overall Survival (mOS); mPFS, Progression-free Survival.

Among the 54 patients, there were 18 (33.33%) survivors, 31 (57.41%) deaths, and 5 (9.26%) lost to follow-up. The missing data was not included in the survival analysis, univariate analysis, or multivariate analysis. The median follow-up time was 71 (rang: 3.03 - 182.97) months. The mOS for all patients was 20.9 months (95% CI 15.7 - 67.6), and the 1-year, 2-year, and 5-year OS rates were 83.39%, 43.22%, and 32.74%, respectively. The mOS for pediatric patients was 15.3 months, with the OS rates at 1-year, 2-year, and 5-year being 78.94%, 24.56%, and 24.56% respectively. The mOS for adult patients was 27.7 months, with the OS rates at 1-year, 2-year, and 5-year being 83.39%, 55.87%, and 38.41% respectively. The mPFS for all patients was 13.7 months (95% CI: 8.83 - 20.08), with the 1-year, 2-year, and 5-year PFS rates were 57.02%, 28.39%, and 21.36%, respectively. The mPFS for pediatric patients was 8.4 months, with the PFS rates at 1-year, 2-year, and 5-year being 40.91%, 17.05%, and 17.05% respectively. Children’s survival is worse than that of patients of all ages.The mPFS for adult patients was 16.5 months, with the PFS rates at 1-year, 2-year, and 5-year being 68.18%, 36.36%, and 24.94% respectively (**Figure 2**, **Table 4**).

**Table 4:** Survival datas.

Survival status	Case(%)
Survival	18(33.33%)
Death	31(57.41%)
Lost to follow-up	5(9.26%)
OS (months)	20.9
1-year OS rate	83.39%
2-year OS rate	43.22%
5-year OS rate	32.74%
PFS (months)	13.7
1-year PFS rate	57.02%
2-year PFS rate	28.39%
5-year PFS rate	21.36%
Adult OS (months)	27.7
1-year OS rate	83.39%
2-year OS rate	55.87%
5-year OS rate	38.41%
Adult PFS (months)	16.5
1-year PFS rate	68.18%
2-year PFS rate	36.36%
5-year PFS rate	24.94%
Child OS (months)	15.3
1-year OS rate	78.94%
2-year OS rate	24.56%
5-year OS rate	24.56%
Child PFS (months)	8.4
1-year PFS rate	40.91%
2-year PFS rate	17.05%
5-year PFS rate	17.05%

mOS, Median Overall Survival (mOS); mPFS, Progression-free Survival.

Univariate Cox regression analysis revealed that tumor volume > 8 cm³(HR 3.04; 95% CI 1.61–7.97; p = 0.024), age 3–17 years (HR 2.27; 95% CI 1.04–4.96; p = 0.039), and age 51–69 years (HR 5.47; 95% CI 1.76 - 17.02; p = 0.003) increased risk of death, while KPS ≥ 70 was the only variable associated with a low risk of mortality (HR 0.39; 95% CI 0.19 - 0.82; p = 0.013). But, Gender, tumor location, tumor invasion, obstructive hydrocephalus,ventricular drainage, surgery, drug treatment did not affect patient survival (**Table 5**).

**Table 5:** Univariate analysis.

Variable	HR	HR.95L	HR.95H	Pvalue
age(3-17)	2.27	1.04	4.96	0.039
age(51-69)	5.47	1.76	17.02	0.003
KPS ≥ 70	0.39	0.19	0.82	0.013
ventricular drainage	1.14	0.39	3.26	0.814
obstructive hydrocephalus	1.97	0.87	4.43	0.103
surgery	0.60	0.14	2.54	0.493
drug treatment	0.87	0.43	1.76	0.690
gender	1.33	0.61	2.89	0.473
tuomor location (midbrain)	2.54	0.56	11.44	0.226
tuomor location (pons)	2.69	0.92	7.84	0.069
invasion	0.45	0.16	1.30	0.141
tumor volume > 8 cm³	3.04	1.16	7.97	0.024

KPS, Karnofsky performance status.

**Figure 3:**
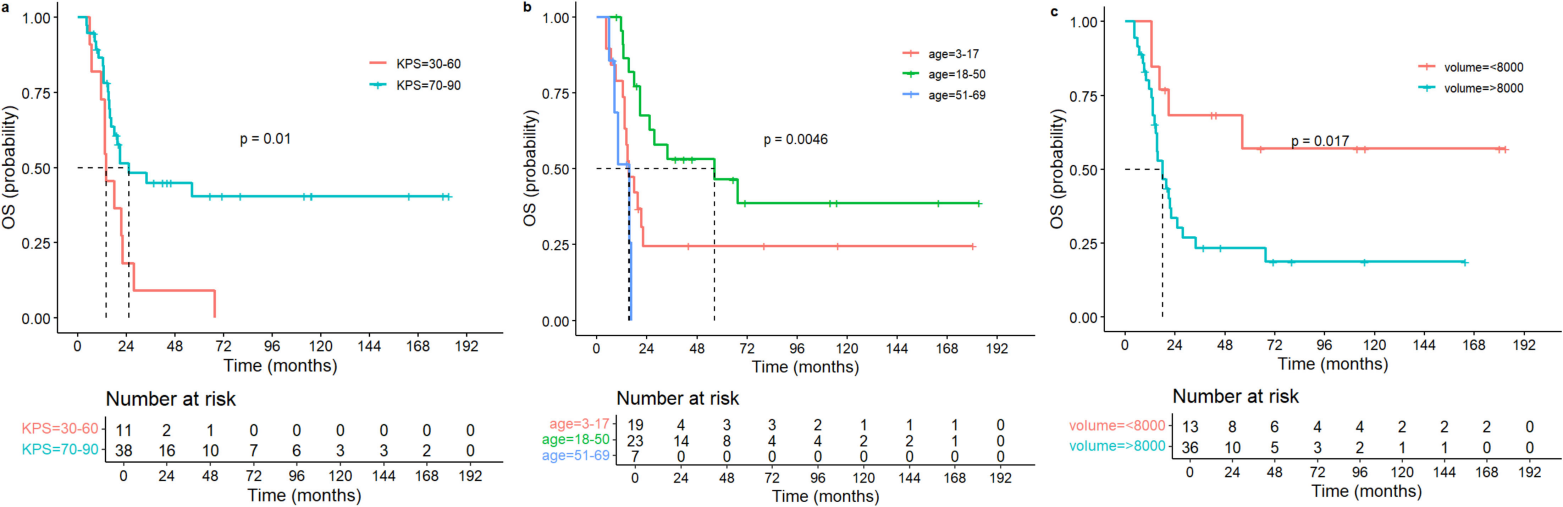
Log-rank analyses: **(a)** Comparison of KPS status with mOS = 25.2 months for KPS ≥ 70 group and mOS = 14.1 months for KPS < 70 group (p = 0.01), **(b)** Comparison of age status with mOS = 15.3 months for 3–17 years group, and mOS = 56.3 months for 18–50 years group, and mOS = 15.7 months for 51–69 years group (p = 0.0046); **(c)** Comparison of tumor volume status with mOS was not reached for volume ≤8cm^3^ group and mOS = 18 months for volume > 8cm^3^ group (p = 0.017). Abbreviation: KPS, Karnofsky performance status; mOS, Median Overall Survival.

Log-rank analysis demonstrated patients 3–17 years and 51–69 years to have a significantly worse median survival than 18–50 years (15.3, 15.7 vs. 56.3 months, p = 0.0046), patients with tumor volume > 8 cm³ have a significantly worse median survival than tumor volume ≤ 8 cm³ (18 vs. NA months, p = 0.017), and patients with KPS < 70 have a significantly worse median survival than ≥70 (14.1 vs. 25.2 months, p = 0.01). And Gender, tumor location, tumor invasion, obstructive hydrocephalus,ventricular drainage, surgery, drug treatment did not affect patient survival (**Figure 3**, **Supplementary Figures 1**, **2**).

**Figure 4:**
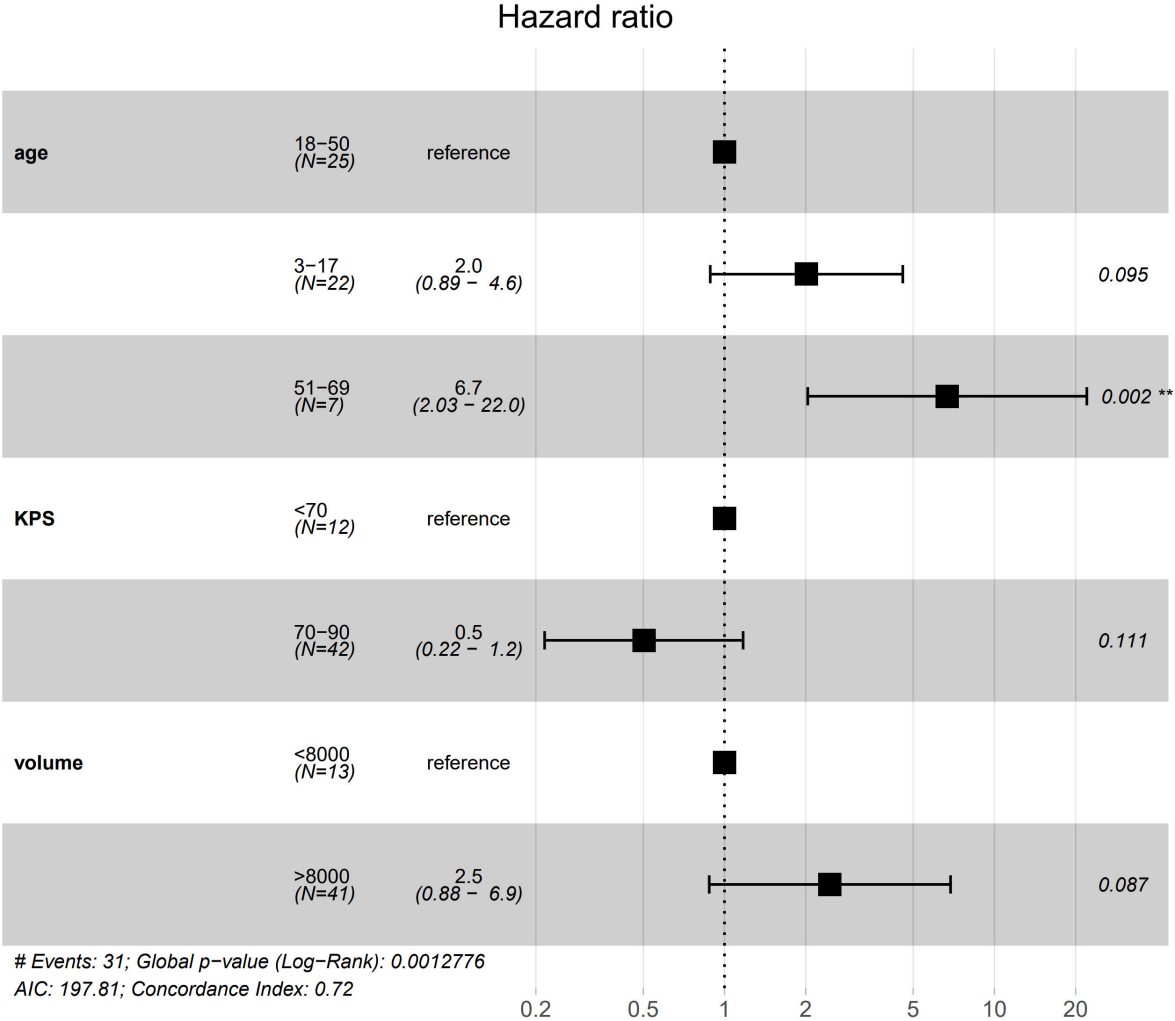
Forest plot of the multivariable Cox proportional-hazards model for overall survival: Adjusted for age, Karnofsky performance status (KPS) and tumor volume, among the three age strata, only the 51–69 years subgroup (versus 18–50 years) remained independently associated with survival (P = 0.002).

Ultimately age, KPS and tumor volume were retained in the multivariate Cox model. Multivariate Cox model analysis revealed that, compared with the 18–50 years, patients aged 51–69 years had an approximately 5.7-fold higher risk of death (HR = 6.69, 95% CI 2.03–22.00;p = 0.002). Age 3–17 years, tumor volume >8 cm³, and KPS 70–90 showed a trend toward association, but the differences did not reach statistical significance (HR = 2.01, p = 0.095; HR = 2.46, p = 0.087; HR = 0.50, p = 0.111) (**Figure 4**, **Table 6**).

**Table 6:** Multivariate analysis.

Variable	HR	HR.95L	HR.95H	P value
age3-17	2.01	0.89	4.58	0.095
age51-69	6.69	2.03	22.00	0.002
KPS70-90	0.50	0.22	1.17	0.111
volume>8cm³	2.46	0.88	6.88	0.087

KPS, Karnofsky performance status.

### Radiotherapy side effects

Radiation-induced cerebral edema occurred in 17 cases (31.48%), and obstructive hydrocephalus after radiotherapy occurred in 4 cases (7.41%). After treatment with mannitol for dehydration, dexamethasone, and Bevacizumab to alleviate cerebral edema, the symptoms were mitigated. Among them, 4 patients with obstructive hydrocephalus underwent ventricular drainage. There were no deaths related to radiotherapy side effects and no radiation necrosis were observed (**Table 7**).

**Table 7:** Radiation adverse reactions/radiation necrosis.

Radiotherapy side effects	N (%)
Radiation-induced brain edema	17(31.48%)
Obstructive hydrocephalus	4(7.41%)

## Discussion

BSG are a rare group of tumours with poor prognosis, and a lack of significantly effective treatment. Due to the localization of brainstem, further limit radical surgery, and lead to difficulties in reaching a molecular pathology and integrated diagnosis (3). And drug treatment is not very effective.

We had studied the clinical characteristics of BSG queues in a mixed population, and confirmed three prognostic indicators to guide treatment decisions. Overall, we found that age, KPS score, and tumor volume may be important predictive factors for prognosis, among which age is an independent prognostic factor. This has been confirmed to varying degrees in many studies in the past (4, 6, 12–16). But the prognostic significance of age, tumor volume, and KPS score in brainstem glioma has been inconsistently reported across studies. This heterogeneity likely reflects methodological limitations inherent to rare disease research, including limited sample sizes, single-institutional biases, and unmeasured confounders such as molecular subtype and extent of resection. Future multi-institutional studies with standardized molecular profiling are warranted to clarify the independent prognostic value of these clinical parameters.

Apart from surgery, radiotherapy is the main treatment method for BSG.Its efficacy is comparable to that of surgery, but with significantly fewer complications. By compared to historical data, Our main findings was that the efficacy of CyberKnife-SRT in treating BSG was slightly better. This effect was consistent across both adult and pediatric patients (**Table 8**). These findings contribute to the evolving landscape of precision radiotherapy for this challenging malignancy characterized by dismal prognosis and limited therapeutic options.

**Table 8:** Comparisontables for various studies.

First author (References)	Type of Research	Publication time	Diagnosis time	patients	age(years)	Tumor type	cases	treatment	mOS(months)
Ius T (3)	meta-analysis	2023	1990-2022	adults	>18	HGG	125	biopsy	6.9
							213	resection	13.6
Doyle J (12)	SEER database	2019	1973–2015	adults	>18	HGG	103	surgery, radiotherapy	11
							15	biopsy	8
							68	subtotal resection	11
							20	total resection	16
Dalmage M (13)	systematic review	2023	NA	children	0.5-17	DIPG	192	biopsy	9.73
Zaghloul MS (14)	Randomized Study	2022	2011-2017	children	2-18	DIPG	85	hypofractionated radiotherapy (39 Gy /13 f)	9.6
							84	hypofractionated radiotherapy (45 Gy /15 f)	8.2
							84	Conventional fractionation (54 Gy/30 f)	8.7
Zhou C (15)	retrospective study	2021	2016-2018	adults	24-93	BSG	50	biopsy, radiotherapy, TMZ	13
							17	conventional radiotherapy	11
Leibetseder, A (16)	retrospective study	2022	2000-2019	adults	18-71	BSG	34	surgery, TMZ, radiotherapy	24.1
Shrieve DC (17)	Clinical controlled trial	1992	1984-1990	children and adults	2-69	BSG	60	hyperfractionated radiotherapy	17.17
Mandell LR (18)	III randomized controlled trial	1999	1992-1996	children and adults	3-21	DIPGs	65	hyperfractionated radiotherapy	8
							67	conventional radiotherapy	8.5
Zaghloul MS (19)	randomized controlled trial	2014	2007-2011	children	<18	DIPG	35	hypofractionated radiotherapy	7.8
Zhang J (20)	retrospectively analyzed	2019	2006-2015	adults	18-70	BSG	18	CyberKnife-SRT	19
Vitanza NA (21)	phase 1 trial	2025	NA	children	2-22	DIPG	21	radiotherapy, B7-H3 CAR T cells	19.8
Huangfu L (22)	phase I clinical trial	2025	NA	children and adults	3-59	BSG	11	surgery, TMZ, Bev,sonodynamic therapy, radiotherapy	11.7
Liu X (23)	Phase IIa clinical trial	2026	2022-2024	children and adultss	NA	HGG	24	Sonodynamic therapy, Stupp regimen	15.6
							39	Stupp regimen	10.8
Qian X (24)	phase I clinical trials	2025	2023	children	5-12	DIPG	9	radiotherapy, oncolytic adenovirus Ad-TD-nsIL12	11.3
Souweidane MM (25)	dose-escalation trial	2025	2012-2022	children	2-21	DIPG	50	radio-immunotheranostic 124I-Omburtamab	15.29
Our study	retrospective study	2025	2009-2024	children and adults	3-69	BSG	54	CyberKnife-SRT, TMZ, surgery	20.9
				adults	18-69		32		27.7
				children	3-17		22		15.3

The survival outcomes observed in our cohort appear comparable to or modestly improved compared with historical benchmarks for sugery in high-grade gliomas (HGG) and DIPG. The mOS for adult patients in our cohort was 27.7 months, and for children patients also had 15.3 months. But they did not experience life-threatening toxicity or adverse reactions. A study from the SEER database for adult patients with high-grade gliomas (HGG) of the brainstem showed that the mOS for patients who underwent biopsy, subtotal resection, and total resection were 8 months, 11 months, and 16 months, respectively (12). Another meta-analysis showed that the mOS after brainstem HGG resection in adults was 13.6 months, and after biopsy was 6.9 months, with a significantly higher incidence of surgical complications, suggesting that it is difficult to recommend surgery instead of biopsy (3). A systematic review showed that the mOS after biopsy for children patients with DIPG aged 0.5 to 17 years was only 9.73 months (13).

In our study, the mOS for children and adults patients was 20.9 months, with a mPFS of 13.7 months. The 1-year and 2-year OS rates were 83.9% and 43.22%, respectively, which are longer than those reported for some other radiotherapy methods. This result was no exception in pediatric patients. Shrieve DC (17) et al. used hyperfractionated radiotherapy to treat children and adults with BSG, with mOS of about 17.17 months, and 1-year and 2-year OS rates of 65% and 38%, respectively. In a phase III randomized controlled trial of children with DIPG, the mOS for conventional radiotherapy was 8.5 months, the mPFS was 6 months, and the 1-year and 2-year OS rates were 30.9% and 7.1%, respectively. The mOS for hyperfractionated radiotherapy was 8 months, the mPFS was 5 months, and the 1-year and 2-year OS rates were 27.0% and 6.7%, respectively (18). All of these were worse than our results. In the study by Zaghloul MS (14) et al, the mOS of hypofractionated radiotherapy and conventional radiotherapy for DIPG children patients were 9.6 and 8.7, respectively. A randomized controlled trial comparing children with DIPG who received conventional radiotherapy or hypofractionated radiotherapy showed that the mOS in the conventional radiotherapy group was 9.5 months, and the mPFS was 7.3 months. The mOS in the hypofractionated radiotherapy group was 7.8 months, and the mPFS was 6.6 months (19). All of these were worse than our results. Similar research findings also indicated the advantages of CyberKnife-SRT for BSG. The study analyzed the survival of 18 adult BSG patients, showing a mOS of 19 months (20). but this study included a relatively small number of cases and did not include pediatric patients.

There was a lack of reliable clinical trial evidence to prove whether there is a synergistic effect of surgery, radiotherapy, chemotherapy, and targeted therapy in BSG treatment. Our study showed that patients who received postoperative radiotherapy as well as those who received temozolomide chemotherapy and/or bevacizumab targeted therapy after radiotherapy had no difference in prognosis compared to those who received radiotherapy alone. This is consistent with the findings of studies by Zhou C (15) et al, who retrospectively analyzed BSG adult patients treated with postoperative radiotherapy and temozolomide chemotherapy, finding a mOS of 13 months, which is worse than our observation. In the study of Leibetseder A (16) et al, a combined treatment approach of surgery, radiotherapy, and chemotherapy did not significantly improve the mOS of adult BSG patients, which was 24.1 months.

In recent years, novel therapeutic methods such as CAR T cells immunotherapy (21), targeted therapy (22), sonodynamic therapy (23), oncolytic virus therapy (24), and radio-immunotheranostic (25) have been introduced in clinical trials. But compared to our research findings, they had not improved the survival of patients with BSG (**Table 8**). These treatment methods need further exploration in prospective clinical trials to improve the poor prognosis of BSG patients. The immunomodulatory effects of high-dose radiotherapy, termed the “abscopal effect, provided rationale for combining SRT with immune checkpoint inhibitors or cellular therapies such as CAR T-cell treatment. Perhaps optimizing CyberKnife-SRT in combination with novel therapeutic methods could bring hope. However, due to the special nature of the brainstem region, this combination method presents unique challenges in terms of neurotoxicity.

In conclusion, CyberKnife-SRT achieved slightly better survival outcomes for BSG compared to surgery and other forms of therapy while offering logistical advantages and potential for dose escalation. However, the inherent limitations of single-arm retrospective data necessitate validation through randomized controlled trials. Future investigations should prioritize molecular stratification, integration of systemic therapies, and quality-of-life endpoints to definitively establish the role of CyberKnife-SRT in this devastating disease.

### Constraint

This study was a small sample, single-center retrospective survey precluded definitive conclusions regarding CyberKnife-SRT superiority over surgery and other forms of therapy; The research subject a heterogeneous group encompassing all brainstem gliomas. Although subgroup analyses were conducted based on age groups, it was not possible to distinguish pathological types, molecular characteristics, and did imaging features. But heterogeneity in histological grade and molecular profiles within our cohort might have influenced outcomes, as H3K27 mutation status, IDH mutation status and MGMT methylation are established prognostic factors in BSG; Furthermore, the integration of systemic treatments was not consistent, which could affect the accuracy of survival analyses. In addition, due to the retrospective nature of the study, telephone follow-ups were primarily used during long-term follow-up, which limited the assessment of late toxicities, particularly radiation-induced necrosis. Such toxicities may not become apparent until several months to years after radiotherapy.

## Conclusion

The efficacy of CyberKnife-SRT for BSG was better than conventional radiotherapy, low fractionation radiotherapy and surgery, and the side effects are controllable.

## Data Availability

The original contributions presented in the study are included in the article/**Supplementary Material**. Further inquiries can be directed to the corresponding author.

## Supplementary material

Supplementary Figure 1 Log-rank analysis of OS after CyberKnife-SRT according to tumor location: **(a)** midbrain, pons, and medulla oblongata; **(b)** medulla oblongata vs pons; **(c)** medulla oblongata vs midbrain; **(d)** midbrain vs pons. Abbreviation: OS, Overall Survival; CyberKnife-SRT, CyberKnife Stereotactic Radiation Therapy.

Supplementary Figure 2 Log-rank analyses of OS after CyberKnife-SRT: **(a)** male versus female, **(b)** patients with versus without obstructive hydrocephalus (OH), **(c)** patients with versus without external ventricular drainage (EVD), **(d)** patients with versus without prior surgery, **(e)** patients with versus without tumor invasion, **(f)** patients with versus without pharmacotherapy. Abbreviation: OS, Overall Survival; CyberKnife-SRT, CyberKnife Stereotactic Radiation Therapy; OH, obstructive hydrocephalus; EVD, external ventricular drainage.
