# Supplementary figures and images for: Evaluation on the efficacy and safety of CyberKnife stereotactic radiotherapy for brainstem gliomas
Source: Front Oncol. 2026 Mar 27;16:1730758. doi: 10.3389/fonc.2026.1730758 (PMC13065695; doi:10.3389/fonc.2026.1730758)

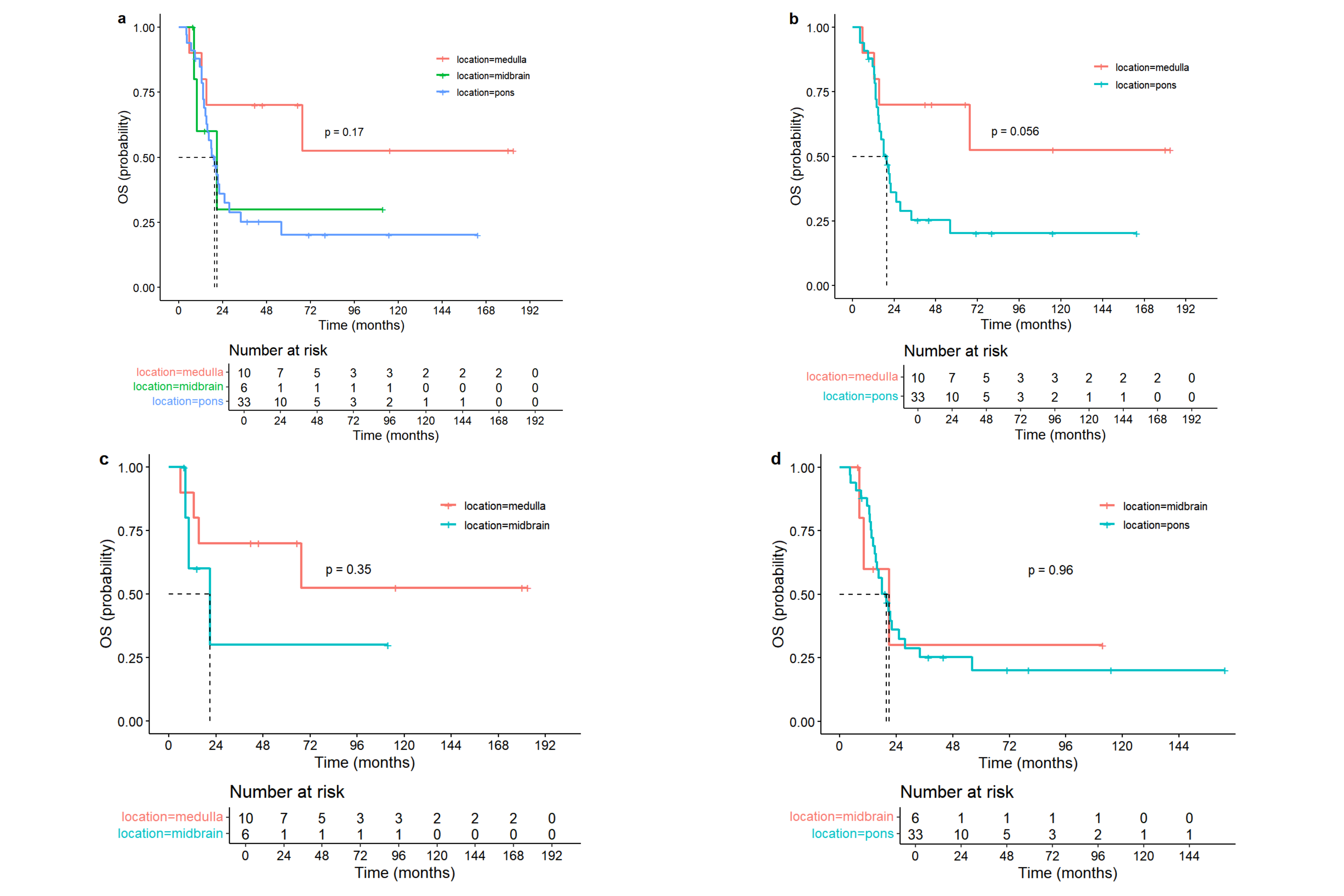

Supplement: Supplementary Figure 1 — Log-rank analysis of OS after CyberKnife-SRT according to tumor location: (a) midbrain, pons, and medulla oblongata; (b) medulla oblongata vs pons; (c) medulla oblongata vs midbrain; (d) midbrain vs pons. Abbreviation: OS, Overall Survival; CyberKnife-SRT, CyberKnife Stereotactic Radiation Therapy. [file Image1.tif]

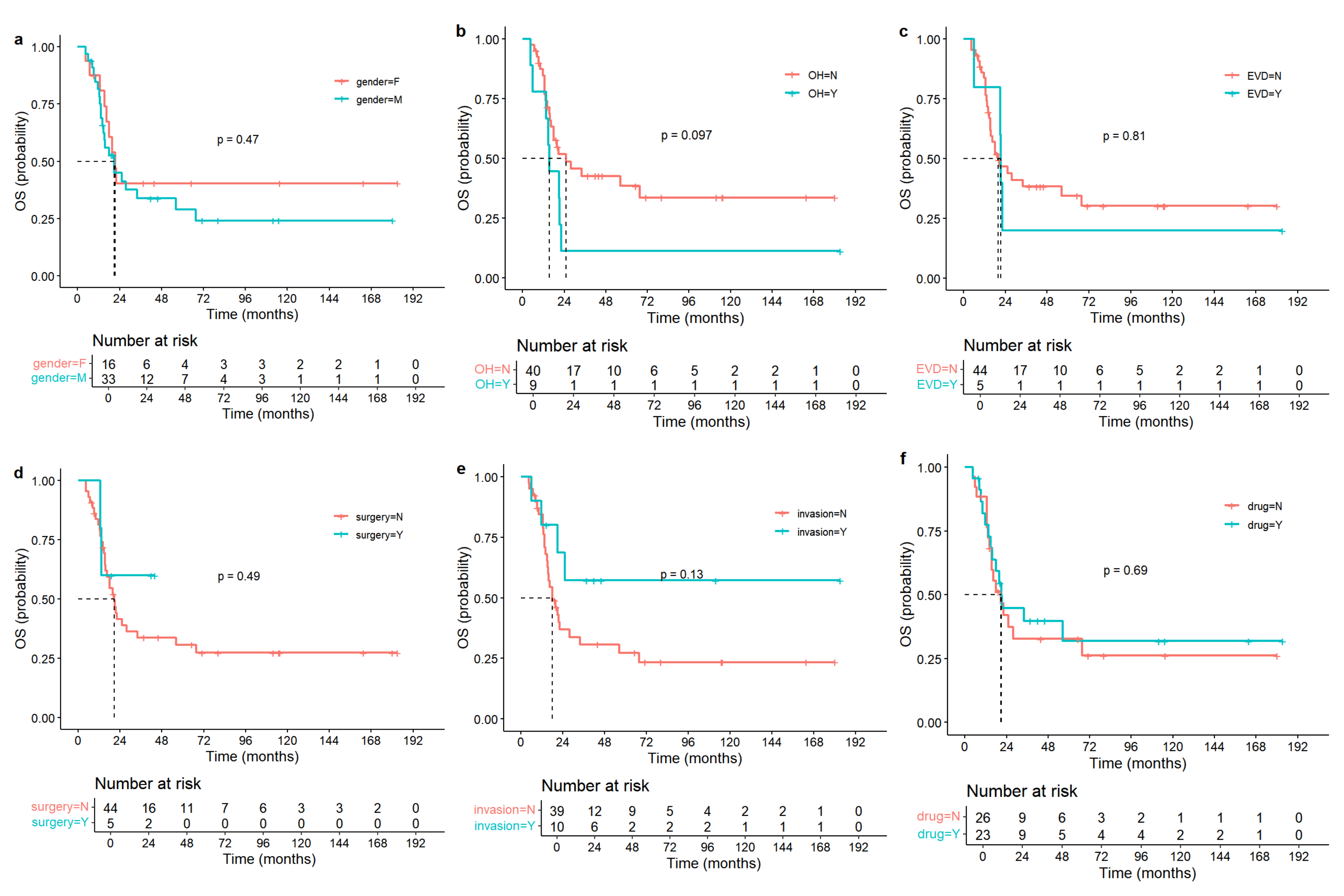

Supplement: Supplementary Figure 2 — Log-rank analyses of OS after CyberKnife-SRT: (a) male versus female, (b) patients with versus without obstructive hydrocephalus (OH), (c) patients with versus without external ventricular drainage (EVD), (d) patients with versus without prior surgery, (e) patients with versus without tumor invasion, (f) patients with versus without pharmacotherapy. Abbreviation: OS, Overall Survival; CyberKnife-SRT, CyberKnife Stereotactic Radiation Therapy; OH, obstructive hydrocephalus; EVD, external ventricular drainage. [file Image2.tif]
